# Effect of platelet activating factor antagonist treatment on gentamicin nephrotoxicity

**DOI:** 10.1155/S096293519200005X

**Published:** 1992

**Authors:** A. Rodriguez-Barbero, E. Bosque, L. Rivas-Cabaero, M. Arévalo, J. M. López-Novoa

**Affiliations:** 1 Departmento de Fisiologia y Farmacologia Facultad de Medicina Universidad de Salamanca Avda, Campo Charro s/n salamanca 37007 Spain; 2 Departmento de Anatomia e Histologia Facultad de Medicina Universidad de Salamanca Avda, Campo Charro s/n salamanca 37007 Spain

## Abstract

To assess whether PAF could be involved in the gentamicin-induced nephrotoxicity, we have studied the effect of PAF antagonist BN-52021 on renal function in rats after gentamicin (GENTA) treatment. Experiments were completed in 21 Wistar rats divided into three groups: group GENTA was injected with gentamicin 100 mg kg^−1^ body wt/day s.c. for 6 days. Group GENTA + BN received gentamicin and BN-52021 i.p. 5 mg kg^−1^ body wt/day. A third group served as control. Rats were placed in meta-bolic cages and plasma creatinine and creatinine clearance were measured daily. GENTA group showed a progressive increase in plasma creatinine, a drop in creatinine clearance and an increase in urinary excretion of *N*-acetyl-*β*-D-glucosaminidase and alkaline phosphatase. GENTA + BN group showed a lesser change in plasma creatinine and a creatinine clearance, but no difference with GENTA group in urinary excretion of NAG and AP were observed. Histological examination revealed a massive cortical tubular necrosis in rats treated with gentamicin, whereas in BN-52021 injected animals tubular damage was markedly attenuated. The present results suggest a role for PAF in the gentamicininduced nephro-toxicity.


Mediators of Inflammation 1, 23–26 (1992)

To assess whether PAF could be involved in the gentamicin-induced nephrotoxicity, we have studied the effect of PAF antagonist BN-52021 on renal function in rats after gentamicin (GENTA) treatment. Experiments were completed in 21 Wistar rats divided into three groups: group GENTA was injected with gentamicin 100 mg $kg^{-1}$ body wt/day s.c. for 6 days. Group GENTA + BN received gentamicin and BN-52021 i.p. 5 mg $kg^{-1}$ body wt/day. A third group served as control. Rats were placed in meta-bolic cages and plasma creatinine and creatinine clearance were measured daily. GENTA group showed a progressive increase in plasma creatinine, a drop in creatinine clearance and an increase in urinary excretion of *N*-acetyl-β-D-glucosaminidase and alkaline phosphatase. GENTA + BN group showed a lesser change in plasma creatinine and a creatinine clearance, but no difference with GENTA group in urinary excretion of NAG and AP were observed. Histological examination revealed a massive cortical tubular necrosis in rats treated with gentamicin, whereas in BN-52021 injected animals tubular damage was markedly attenuated. The present results suggest a role for PAF in the gentamicin-induced nephro-toxicity.

**Key words:** Gentamicin, Kidney, Nephrotoxicity, Platelet activating factor, Proximal tubule, Renal function

### Effect of platelet activating factor antagonist treatment on gentamicin nephrotoxicity

A. Rodriguez-Barbero,[1] E. Bosque,[1] L. Rivas-Cabañero,[1] M. Arévalo,[2] and J.M. López-Novoa[1,CA]

[1]Departmento de Fisiologia y Farmacologia, and [2]Departmento de Anatomia e Histologia, Facultad de Medicina, Universidad de Salamanca, Avda, Campo Charro s/n, 37007-Salamanca, Spain

[CA] Corresponding Author

### Introduction

Gentamicin is an aminoglycoside frequently used in the treatment of gram-negative infection. One of its main side effects is nephrotoxicity, even at the lower therapeutical doses.[1] Gentamicin is specifically reabsorbed by proximal tubular cells,[2] and accumulated into the lysosomes, inducing $PIP_2$-enriched phospholipidosis[1] and thus, causes cellular necrosis. However, although gentamicin-induced nephrotoxicity is mainly tubular, and cellular necrosis confined to cells of the nephron segment where gentamicin is absorbed,[3] chronic treatment with gentamicin has been reported to induce decreases in glomerular filtration rate (GFR).[4]

Platelet activating factor (PAF) has been shown to be produced by the kidney[5,6] and to be involved in the reduction of GFR observed in experimental models of acute renal failure (ARF).[7,8]

To assess whether PAF could be involved in the gentamicin-induced nephrotoxicity, we have studied in rats after gentamicin treatment, the effect on renal function and morphology of inhibiting the interaction of PAF with its receptor using a specific antagonist of PAF, BN-52021.[9]

### Methods

Experiments were completed in 21 Wistar rats weighing about 250 g. The animals were placed into individual metabolic cages for 4 days, and urine, free of food and faeces, was collected daily into graduate cylinders under water-equilibrated mineral oil, 2 days before and 6 days after starting gentamicin treatment. On the last 2 days, urine was collected on ice to determine enzymatic urine activity. A blood sample (150 μl) was taken from the caudal vein before and daily after starting gentamicin treatment. Rats were divided into three experimental groups:

Group I. Control rats received a daily s.c. injection of 0.5 ml isotonic saline solution and i.p. injection of 0.5 ml of saline solution, for 6 days.

Group II. This group was injected with gentamicin-sulphate 100 mg $kg^{-1}$ body wt/day s.c. in 0.5 ml and saline solution 0.5 ml i.p., for 6 days.

Group III. This group received gentamicin in the same conditions as group II and BN-52021 i.p. 5 mg $kg^{-1}$ body wt/day in 0.5 ml saline solution, for 6 days.

The last day, rats were slightly anaesthetized with ether, and 5 ml of blood was taken from the abdominal aorta into heparinized syringes. The kidneys were removed and small pieces from one kidney were placed in buffered 10% formalin and processed for subsequent examination (in a blinded fashion) by light microscopy. In the second kidney, the cortex was isolated and gentamicin extracted from renal cortex by NaOH digestion according to

© 1992 Rapid Communications of Oxford Ltd the method of Brown *et al.*,[10] and its content was determined by a fluorescence polarization using the Cobas Fara II system (Roche).

Morphological changes were analysed in a blind way by a pathologist (M.A.) and scored with a semi-quantitative scale evaluating the changes more frequently found in acute renal failure. Higher scores represent more severe damage (maximum score per tubule was ten with points given for brush-border loss (1 point), cell membrane bleb formation (1 or 2 points), interstitial oedema (1 point), cell necrosis (1 or 2 points), cytoplasmic vacuolization (1 point), tubular lumen obstruction (1 or 2 points) and lympho-monocyte infiltration (1 point).

Plasma and urine creatinine were determined by a colorimetric method based on the Jaffé reaction. *N*-acetyl-*β*-D-glucosaminidaze (NAG) was measured by a spectrofluorimetric method[11] and alkaline phosphatase (AP) by a spectrophotometric method (Boehringer Mannheim).[12]

Gentamicin sulphate was a gift of Antibioticos Farma, Madrid, Spain; BN-52021 was obtained from Institut Henri Beaufour, Les Ulis, France. All the other reagents were purchased from Sigma. Data are expressed as mean $\pm$ SEM. One or two ways analysis of variance were used to compare the effects of drug treatment between groups. A $p$ value lower then 0.01 was considered statistically significant.

### Results

In rats that received gentamicin, renal function deteriorated over 5 days, with a two-fold increase in plasma creatinine and a drop in creatinine clearance (Figs 1 and 2). In addition these animals showed an increase in urinary excretion of *N*-acetyl-*β*-D-glucosaminidase and alkaline phosphatase (Table 1). Rats treated with gentamicin and BN-52021 showed a lower increase in plasma creatinine and a lower decrease in creatinine clearance (Figs 1 and 2). However no differences in urinary excretion of NAG and AP were observed between BN-52021 treated and untreated animals.

The renal cortical gentamicin concentration of rats injected with gentamicin plus BN-52021 ($375 \pm 2$ μg/g) tended to be higher than in those injected with gentamicin alone ($310 \pm 43$ μg/g; $p =$ NS). Gentamicin was undetectable in control rats.

Light microscopy examination (Table 2) revealed patchy cell necrosis in more than 85% of the proximal tubules in kidneys of rats injected with gentamicin. In addition, tubular lumen was freqently filled with hyaline casts or heterogeneous cellular debris. In contrast, rats treated with BN-52021, manifest cell necrosis was observed in less than 25% of cells, although most proximal cells showed signs of cell degeneration, as cytoplasmic vacuolization, membrane blebs and loss of the brush border. Tubular lumens obstructed with hyaline material were rare. No glomerular damage was observed in gentamicin-treated rats receiving or not BN-52021. Kidneys from the control rats revealed neither cellular necrosis nor tubular obstruction.

### Discussion

From the data shown above, it can be deduced that the treatment with gentamicin induces a reduction in glomerular filtration rate, manifested

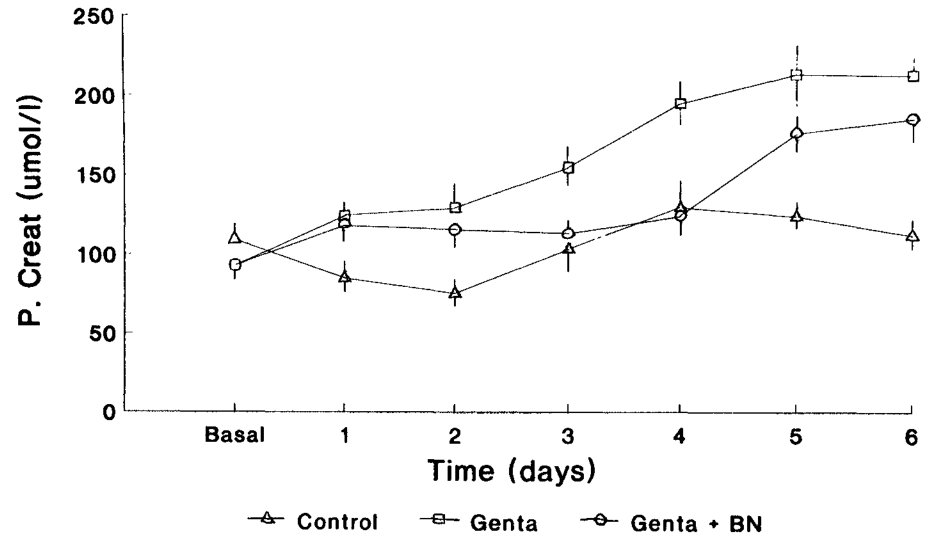

FIG. 1. Time course of plasma creatinine after gentamicin injection in the three groups of rats. Data represents the mean $\pm$ SEM. All the curves are significantly different to each other ($p < 0.01$; two ways analysis of variance).

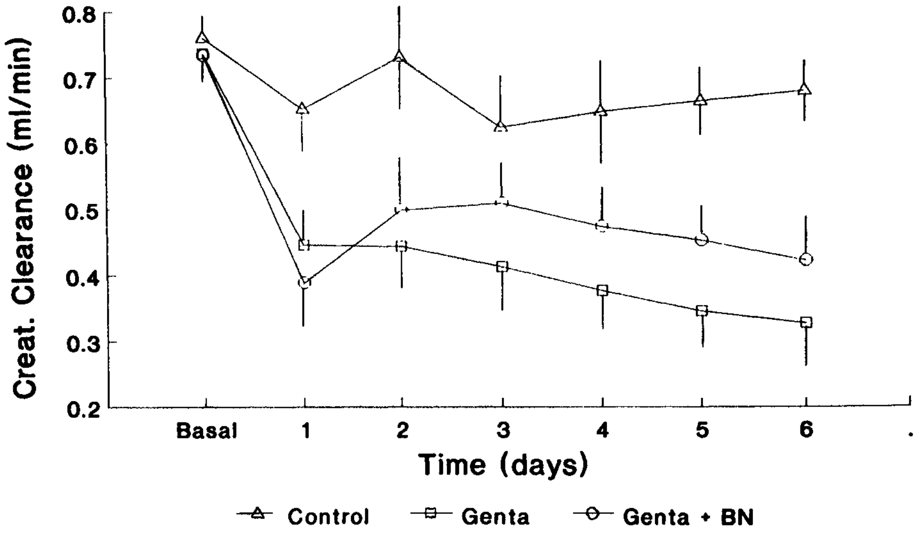

FIG. 2. Time course of creatinine clearance after gentamicin injection in the three groups of rats. Data represents the mean ± SEM. All the curves are significantly different to each other ($p < 0.01$; two ways analysis of variance).

**Table 1**. Urinary excretion of NAG and AP

| | NAG (U/d) | AP (nKat/d) |
|---|---|---|
| CONT | 70.78 ± 20.60 | 0.048 ± 0.02 |
| GENT | 673.22 ± 109.1* | 2.955 ± 1.59* |
| G + BN | 621.06 ± 80.8* | 1.827 ± 0.90* |

Data are means ± SEM.* $p < 0.01$ with control group (one way analysis of the variance). Abbreviations: CONT: control group; GENT: group treated with gentamicin (100 mg/kg/day); G + BN: group treated with gentamicin and BN-52021 (5 mg/kg/day). NAG: *N*-Acethyl-β-D-glucosaminidase; AP: alkaline phosphatase.

by the progressive reduction of the creatinine clearance, and the increase in plasma creatinine. In addition, these animals showed a marked increase in the urinary excretion of NAG and AP. NAG is a lysosomal enzyme, and its increased urinary excretion reflects the lysosomal damage induced by gentamicin.[13] AP is a brush border-associated enzyme, and its increased urinary excretion reflects the cellular necrosis induced by the drug. The data agree with the effect of gentamicin on renal function reported in previous studies.[2,3]

When the rats, in addition to gentamicin, were treated with BN-52021, a PAF antagonist, the increase in plasma creatinine was slower than that shown by gentamicin treated, BN-untreated rats. According to these results, the decrease in creatinine clearance was less marked in BN-treated than in untreated rats. Previous studies from our laboratory have also demonstrated a protection of the renal function by PAF antagonists in several models of acute renal insufficiency.[7,8] Thus, it could be suggested that gentamicin treatment induces a release of PAF by some kind of renal cells, and this PAF could act on renal resistance vessels and/or mesangial cells, where receptors for PAF exist, thus inducing a decrease in GFR, and renal blood flow.

**Table 2**. Scores of tubular damage among the different groups

| | CONT | GENTA | GENTA + BN |
|---|---|---|---|
| Brush-border loss | 0.08 ± 0.001 | 1°* | 0.80 ± 0.12#* |
| Blebs | 0.08 ± 0.001 | 2°* | 1.08 ± 0.13#* |
| Interstitial oedema | 0.00 ± 0.00 | 0.22 ± 0.12* | 0.08 ± 0.02# |
| Cytoplasmic vacuolization | 0.22 ± 0.05 | 1°* | 0.89 ± 0.013#* |
| Necrosis | 0.03 ± 0.00 | 1.81 ± 0.13* | 1.04 ± 0.16#* |
| Tubular obstruction | 0.00 ± 0.00 | 1.62 ± 0.14* | 0.25 ± 0.14#* |
| Infiltration | 0.00 ± 0.00 | 0.40 ± 0.11* | 0.10 ± 0.005#* |
| TOTAL | 0.41 ± 0.05 | 8.05 ± 0.40* | 4.27 ± 0.35#* |

Abbreviations: CONT: control group; GENTA: group treated with Gentamicin (100 mg/kg/day); GENTA + BN: group treated with gentamicin and BN-52021 (5 mg/kg/day). °As the cells were too damaged, these concepts were impossible to be scored. A maximum value was given in order to obtain the total score. Data are mean ± SEM; *statistically significant differences ($p < 0.05$, one way analysis of the variance) with respect to control rats; #statistically significant differences ($p < 0.05$, one way analysis of the variance with respect to GENTA-treated rats.

Although it cannot be deduced from the present experiments, PAF is probably originated in the glomerular endothelial and mesangial cells. In this regard, we have previously demonstrated that glomeruli from rats with acute renal failure show an increased production of PAF.[14]

Urinary excretion of NAG and AP, a marker of tubular damage,[13] was similar in gentamicin-treated rats, with or without treatment with BN-52021, thus suggesting that tubular damage was similar in both groups of rats. However, morphological studies revealed that the severity of proximal cell necrosis was greatly attenuated by BN-52021. PAF does not seem to act directly on tubular cells, since there are not receptors in these cells.[15] Thus, the possible mechanism by which BN-52021 attenuated cell necrosis ameliorates the PAF-mediated gentamicin-induced renal ischemia that probably superimposes the direct tubular action of gentamicin.

Cortical levels of gentamicin were also similar in both groups of animals. Thus, it can be deduced that the protective effect of BN-52021 against gentamicin nephrotoxicity was not mediated by an inhibition of cortical cell accumulation of gentamicin.

In summary, the present results demonstrate that BN-52021 blunted the fall in GFR and greatly attenuated the severity of proximal cell necrosis induced by gentamicin. The data suggest a role for PAF in the gentamicin-induced nephrotoxicity. However further experiments, using other structurally-unrelated PAF antagonists, as well as measuring the production of PAF by kidney structures after treatment with gentamicin are necessary to confirm a role of PAF in gentamicin-induced nephrotoxicity.

### References

1. Kaloyanides GJ. Metabolic interaction between drugs and renal tubulointerstitial cells: Role in nephrotoxicity. *Kidney Int* 1991; **39:** 531–540.
2. Josepovitz C, Pastoriza-Muñoz E, Timmerman D, *et al.* Inhibition of gentamicin uptake in rat renal cortex *in vivo* by aminoglycosides and organic polications. *J Pharmacol Exp Ther* 1982; **223:** 314–321.
3. Kaloyanides GJ, Pastoriza-Muñoz E. Aminoglycoside nephrotoxicity. *Kidney Int* 1980; **18:** 571–582.
4. Schor N, Ichikawa I, Rennke HG, *et al.* Pathophysiology of altered glomerular function in aminoglycoside-treated rats. *Kidney Int* 1981; **19:** 288–296.
5. Pirotzki E, Bidault J, Burtin C, *et al.* Release of platelet activating factor, slow reacting substance and vasoactive amines from isolated rat kidneys. *Kidney Int* 1984; **25:** 404–410.
6. Camussi G. Potential role of platelet activating factor in renal pathophysiology. *Kidney Int* 1986; **29:** 469–477.
7. Lopez-Farré A, Gomez-Garre DN, Bernabeu F, *et al.* Platelet activating factor mediates glycerol-induced acute renal failure in the rat. *Clin Sci* 1990, **79:** 551–558.
8. Lopez-Farré A, Bernabeu F, Gómez-Garre D, *et al.* Platelet activating factor antagonists treatment protects against postischemic acute renal failure in rats. *J Pharmacol Exp Ther* 1990; **253:** 328–333.
9. Braquet P. The gikgolides: potent platelet activating factor antagonists isolated from *Ginkgo Biloba* L.: chemistry, pharmacology and clinical applications. *Drugs Future* 1987; **12:** 643–699.
10. Brown SA, Sugimoto K, Smith GG, *et al.* Improved sodium hydroxide digestion method without homogenization for extraction of gentamicin from renal tissue. *Antimicrob Agents Chemother* 1988; **32:** 595–597.
11. Robinson D, Thorpe R. Fluorescent assay of alpha-L-fucosidase. *Clin Chim Acta* 1974; **55:** 65–69.
12. Bretaudiere JP, Spillman T. Alkanine phosphatases. In: Bergmeyer HU, ed. *Methods in enzymatic analysis, 3rd ed.* Weinheim: Verlag Chemie, 1984; 75–82.
13. Gibey RE, Dupond JL, Albor D, *et al.* Predictive value of urinary *N*-acetyl-$\beta$-D-glucosaminidase (NAG), alanine aminopeptidase and $\beta$-2-microglobulin in evaluating nephrotoxicity of gentamicin. *Clin Chim Acta* 1981; **116:** 25–34.
14. Lopez-Farré, Torralbo M, López-Novoa JM. Glomeruli from ischemic rat kidneys produce increased amounts of platelet activating factor. *Biochem Biophys Res Commun* 1988; **152:** 129–135.
15. Braquet P, Touqui L, Shen TY, *et al.* Perspectives in platelet activating factor research. *Pharmacol Rev* 1987; **39:** 97–145.

ACKNOWLEDGEMENTS. This work has been supported in part by a grant from DGICYT (PM-88-0013-002). We acknowledge the cooperation of Dr. Garcia-Bastos with the gentamicin determination and A. Perez with the histological preparations. We also thank Dr. P. Braquet, Institut Henri Beaufour, Les Ulis, France, for the gift of BN-52021, and Dr. MA de Obeso, Antibioticos Farma, Madrid, Spain, for the gift of gentamicin.

**Received 10 October 1991; accepted in revised form 14 November 1991**
